# It is time to integrate models across disciplines: a commentary on Krüger et al. (2022)

**DOI:** 10.1007/s00426-024-01930-3

**Published:** 2024-03-02

**Authors:** Christian Seegelke, Tobias Heed

**Affiliations:** 1 Department of Psychology, University of Salzburg, Hellbrunner Straße 34, 5020 Salzburg, Austria; 2 Centre for Cognitive Neuroscience, University of Salzburg, Salzburg, Austria

In their target article, Krüger et al. (2022) provide a comprehensive review of the many motor imagery facts that have been gathered in recent years. One point that strikes us is a lack of convergence between different theoretical frameworks. As is currently the case in many areas of cognitive (neuro)science and psychology, what we have is a list of findings that we can sort according to essentially atheoretical criteria, such as “representations” and “experience and expertise.” Yet, some reliable phenomena are beginning to emerge from these collections. For example, the shared assumption across many accounts in sensorimotor control that sensory predictions play a pivotal role in the planning and control of movements may encourage new efforts to mediate between models, approaches, or frameworks that are currently separate from each other. Such thoughts appear in the review, e.g., in brief comments on the (supposed) incompatibility between ideomotor theory and forward models but are not developed further.

Theories and frameworks usually emerge in circumscribed fields and focus on issues that are relevant to the respective community. Accordingly, they often lack the ability to directly address questions currently asked in other fields. Consequently, theories are often mentioned but not really adopted and extended by other fields. We would like to acknowledge two recent examples––originating from the field of motor learning––that have attempted to integrate findings from different domains into a unified account. Of course, our selection reflects a subjective choice and is not meant as an exhaustive list. First, phenomena from different learning paradigms (classic conditioning, episodic memory, instrumental learning, and motor adaptation) have been connected within an overarching formal Bayesian model which puts the inference of context at the heart of learning (Heald et al., 2023). Second, some puzzling findings in using cues for context switching in motor adaptation (Gippert et al., 2023; Howard et al., 2020) have been explained by falling back on general characteristics of associative learning (Avraham et al., 2022). Independent of whether these works turn out to hold against further experimental scrutiny, they demonstrate how reaching beyond the concepts of a circumscribed field can stimulate new and integrative theorizing.

We envision that motor imagery, too, could serve as a hook for such integration across theories and fields. In other words, the motor imagery phenomena summarized by Krüger et al. (2022) could trigger more dedicated theory development.

For instance, as pointed out by the authors, ideomotor theory accounts only for goal, but not movement-related sensory information. The theory is based on the idea that the prediction of sensory goal states triggers the associated movements that realize them. This stands in contrast to the sensory predictions output by a forward model, which also encompass the sensations experienced during movement execution to achieve that goal state. It seems straightforward to expand the concept of goal state representations to integrate movement-related sensory information into this associative conglomerate. Notably, a debate about the relationship between goal states and movement detail exists also in sensorimotor neuroscience, cast as a (potential) dichotomy of action selection vs. action specification (Cisek & Kalaska, 2010). There is no consensus about whether multiple possible actions are present in brain activity only with respect to their attainable goal or whether also the respective underlying, fully specified movement plans are available for each potential action (Cisek & Kalaska, 2005; Dekleva et al., 2018). Could body-specific influences on motor imagery phenomena––which suggest the existence of fine-grained, movement-related details in thought (e.g., de Lange et al., 2006; Willems et al., 2009)––help sharpen theoretical concepts and stimulate attempts to bridge between these theories?

Similarly, motor imagery research might help explaining unresolved issues in the influential optimal feedback control (OFC) theory, a computational motor control framework (Scott, 2016; Shadmehr & Krakauer, 2008; Todorov & Jordan, 2002). The OFC theory emphasizes inherent links between perception and action and posits that the brain continually estimates the state of body and world by combining sensory feedback from different modalities and predictions about the sensory consequences of the motor command (Miall & Wolpert, 1996; Wolpert et al., 1995). One important aspect of OFC is that responses to potential movement perturbations are set prospectively, with varying settings across the time of the movement (Diedrichsen et al., 2010; Franklin & Wolpert, 2011). Yet, these models lack explicit integration of body-related processing (Martel et al., 2016; Medendorp & Heed, 2019). Could one exploit the fact that motor imagery removes real feedback and leaves only internally computed predictions to tease apart some of the concepts that are currently underspecified in OFC? Moreover, OFC models do not account for asynchronous processing delays across different modalities in the context of rapid movement corrections (Cluff et al., 2015; Oostwoud Wijdenes & Medendorp, 2017). For example, some authors have postulated that multisensory integration in online control uses both sensory variability and temporal delays (Crevecoeur et al., 2016); others, in contrast, have proposed that sensory feedback is initially processed in modality-specific channels (Keyser et al., 2023). From a very different but just as relevant angle, theories of body models emphasize the critical role of multisensory integration, as is well illustrated by the famous rubber hand and Pinocchio nose illusions (Botvinick & Cohen, 1998; Lackner, 1988), among many others. Might the constraints that human imagery abilities place on its implementation help furnish new ideas about how to link the currently disjunct areas of OFC, body models, and multisensory integration?

Finally, motor imagery research might advance theoretic accounts on the neurophysiological basis of sensorimotor control. Research on the neural dynamics underlying the control of movement based on multielectrode array recordings in nonhuman primates has fostered the idea that primary motor cortex can be adequately characterized from a dynamical systems perspective (Shenoy et al., 2013). Accordingly, preparatory activity involves setting an initial neural state specific to the upcoming movement, from which the movement will then mechanistically evolve. In support of this view, planning, but not executing, different follow-through movements allows simultaneous learning of opposing perturbations over the same movement (Sheahan et al. 2016). Importantly, merely imagining such follow-throughs also resulted in substantial learning (Sheahan et al. 2018), suggesting that motor imagery evokes different neural states for the same physical states, from which differentiated motor learning can emerge.

In summary, the rich, multisensory cognitive content elicited by motor imagery poses multiple challenges to existing theories, clearly indicating that we have not yet understood how body representation and control really work. At the same time, these challenges could foster new efforts to extend and bridge between currently isolated theories and, thus, foster true scientific advance.

## References

[CR1] Avraham, G., Taylor, J. A., Breska, A., Ivry, R. B., & McDougle, S. D. (2022). Contextual effects in sensorimotor adaptation adhere to associative learning rules. *eLife,**11*, e75801. 10.7554/eLife.75801 36197002 10.7554/eLife.75801 PMC9635873

[CR2] Botvinick, M., & Cohen, J. (1998). Rubber hands “feel” touch that eyes see. *Nature,**391*, 756. 10.1038/35784 9486643 10.1038/35784

[CR3] Cisek, P., & Kalaska, J. F. (2005). Neural correlates of reaching decisions in dorsal premotor cortex: specification of multiple direction choices and final selection of action. *Neuron,**45*(5), 801–814. 10.1016/j.neuron.2005.01.027 15748854 10.1016/j.neuron.2005.01.027

[CR4] Cisek, P., & Kalaska, J. F. (2010). Neural mechanisms for interacting with a world full of action choices. *Annual Review of Neuroscience,**33*(1), 269–298. 10.1146/annurev.neuro.051508.135409 20345247 10.1146/annurev.neuro.051508.135409

[CR5] Cluff, T., Crevecoeur, F., & Scott, S. H. (2015). A perspective on multisensory integration and rapid perturbation responses. *Vision Research,**110*, 215–222. 10.1016/j.visres.2014.06.011 25014401 10.1016/j.visres.2014.06.011

[CR6] Crevecoeur, F., Munoz, D. P., & Scott, S. H. (2016). Dynamic multisensory integration: somatosensory speed trumps visual accuracy during feedback control. *Journal of Neuroscience,**36*(33), 8598–8611. 10.1523/JNEUROSCI.0184-16.2016 27535908 10.1523/JNEUROSCI.0184-16.2016 PMC6601898

[CR7] de Lange, F. P., Helmich, R. C., & Toni, I. (2006). Posture influences motor imagery: An fMRI study. *NeuroImage,**33*(2), 609–617. 10.1016/j.neuroimage.2006.07.017 16959501 10.1016/j.neuroimage.2006.07.017

[CR8] Dekleva, B. M., Kording, K. P., & Miller, L. E. (2018). Single reach plans in dorsal premotor cortex during a two-target task. *Nature Communications,**9*(1), 3556. 10.1038/s41467-018-05959-y 30177686 10.1038/s41467-018-05959-y PMC6120937

[CR9] Diedrichsen, J., Shadmehr, R., & Ivry, R. B. (2010). The coordination of movement: Optimal feedback control and beyond. *Trends in Cognitive Sciences,**14*(1), 31–39. 10.1016/j.tics.2009.11.004 20005767 10.1016/j.tics.2009.11.004 PMC4350769

[CR10] Franklin, D. W., & Wolpert, D. M. (2011). Computational mechanisms of sensorimotor control. *Neuron,**72*, 425–442. 10.1016/j.neuron.2011.10.006 22078503 10.1016/j.neuron.2011.10.006

[CR11] Gippert, M., Leupold, S., Heed, T., Howard, I. S., Villringer, A., Nikulin, V. V., & Sehm, B. (2023). Prior movement of one arm facilitates motor adaptation in the other. *Journal of Neuroscience*. 10.1523/JNEUROSCI.2166-22.2023 37160362 10.1523/JNEUROSCI.2166-22.2023 PMC10254980

[CR12] Heald, J. B., Lengyel, M., & Wolpert, D. M. (2023). Contextual inference in learning and memory. *Trends in Cognitive Sciences,**27*(1), 43–64. 10.1016/j.tics.2022.10.004 36435674 10.1016/j.tics.2022.10.004 PMC9789331

[CR13] Howard, I. S., Franklin, S., & Franklin, D. W. (2020). Asymmetry in kinematic generalization between visual and passive lead-in movements are consistent with a forward model in the sensorimotor system. *PLoS ONE,**15*(1), e0228083. 10.1371/journal.pone.0228083 31995588 10.1371/journal.pone.0228083 PMC6988934

[CR14] Keyser, J., Medendorp, W. P., Oostwoud Wijdenes, L., & Selen, L. P. J. (2023). Late integration of vision and proprioception during perturbed reaches. *Journal of Neurophysiology,**129*(6), 1282–1292. 10.1152/jn.00324.2022 37073978 10.1152/jn.00324.2022

[CR15] Krüger, B., Hegele, M., & Rieger, M. (2022). The multisensory nature of human action imagery. *Psychological Research Psychologische Forschung*. 10.1007/s00426-022-01771-y 36441293 10.1007/s00426-022-01771-y PMC11315721

[CR16] Lackner, J. R. (1988). Some proprioceptive influences on the perceptual representation of body shape and orientation. *Brain,**111*(2), 281–297. 10.1093/brain/111.2.281 3378137 10.1093/brain/111.2.281

[CR25] Martel, M., Cardinali, L., Roy, A. C., & Farnè, A. (2016). Tool-use: An open window into body representation and its plasticity. *Cognitive Neuropsychology, 33*, 82–101. 10.1080/02643294.2016.1167678 27315277 10.1080/02643294.2016.1167678 PMC4975077

[CR26] Medendorp, W. P., & Heed, T. (2019). State estimation in posterior parietal cortex: Distinct poles of environmental and bodily states. *Progress in Neurobiology, 183*, 101691. 10.1016/j.pneurobio.2019.101691 31499087 10.1016/j.pneurobio.2019.101691

[CR17] Miall, R. C., & Wolpert, D. M. (1996). Forward models for physiological motor control. *Neural Networks,**9*(8), 1265–1279. 10.1016/S0893-6080(96)00035-4 12662535 10.1016/S0893-6080(96)00035-4

[CR18] OostwoudWijdenes, L., & Medendorp, W. P. (2017). State estimation for early feedback responses in reaching: Intramodal or multimodal? *Frontiers in Integrative Neuroscience*. 10.3389/fnint.2017.00038 10.3389/fnint.2017.00038 PMC5742230 29311860

[CR19] Scott, S. H. (2016). A functional taxonomy of bottom-up sensory feedback processing for motor actions. *Trends in Neurosciences,**39*(8), 512–526. 10.1016/j.tins.2016.06.001 27378546 10.1016/j.tins.2016.06.001

[CR20] Shadmehr, R., & Krakauer, J. W. (2008). A computational neuroanatomy for motor control. *Experimental Brain Research,**185*(3), 359–381. 10.1007/s00221-008-1280-5 18251019 10.1007/s00221-008-1280-5 PMC2553854

[CR27] Sheahan, H. R., Franklin, D. W., & Wolpert, D. M. (2016). Motor planning, not execution, separates motor memories. *Neuron, 92*(4), 773–779. 10.1016/j.neuron.2016.10.017 27817979 10.1016/j.neuron.2016.10.017 PMC5167294

[CR28] Sheahan, H. R., Ingram, J. N., Žalalytė, G. M., & Wolpert, D. M. (2018). Imagery of movements immediately following performance allows learning of motor skills that interfere. *Scientific Reports, 8*, 14330. 10.1038/s41598-018-32606-9 30254381 10.1038/s41598-018-32606-9 PMC6156339

[CR21] Shenoy, K. V., Sahani, M., & Churchland, M. M. (2013, July 10). *Cortical Control of Arm Movements: A Dynamical Systems Perspective* [Review-article]. Http://Dx.Doi.Org/10.1146/Annurev-Neuro-062111-150509. http://www.annualreviews.org/eprint/487Q4yXtksgwQNDR3NFW/full/10.1146/annurev-neuro-062111-150509 10.1146/annurev-neuro-062111-150509 23725001

[CR22] Todorov, E., & Jordan, M. I. (2002). Optimal feedback control as a theory of motor coordination. *Nature Neuroscience*. 10.1038/nn963 12404008 10.1038/nn963

[CR23] Willems, R. M., Toni, I., Hagoort, P., & Casasanto, D. (2009). Body-specific motor imagery of hand actions: Neural evidence from right- and left-handers. *Frontiers in Human Neuroscience,**3*, 39. 10.3389/neuro.09.039.2009 19949484 10.3389/neuro.09.039.2009 PMC2784680

[CR24] Wolpert, D. M., Ghahramani, Z., & Jordan, M. I. (1995). An internal model for sensorimotor integration. *Science,**269*(5232), 1880–1882. 7569931 10.1126/science.7569931

